# Supplementary material for: Genetic characterization of a unique neuroendocrine transdifferentiation prostate circulating tumor cell-derived eXplant model
Source: Nat Commun. 2020 Apr 20;11:1884. doi: 10.1038/s41467-020-15426-2 (PMC7171138; doi:10.1038/s41467-020-15426-2)
Supplement: Supplementary file 1 — Supplementary Information [file 41467_2020_15426_MOESM1_ESM.pdf]

Supplementary Information

Supplementary Figure Legends – Final version

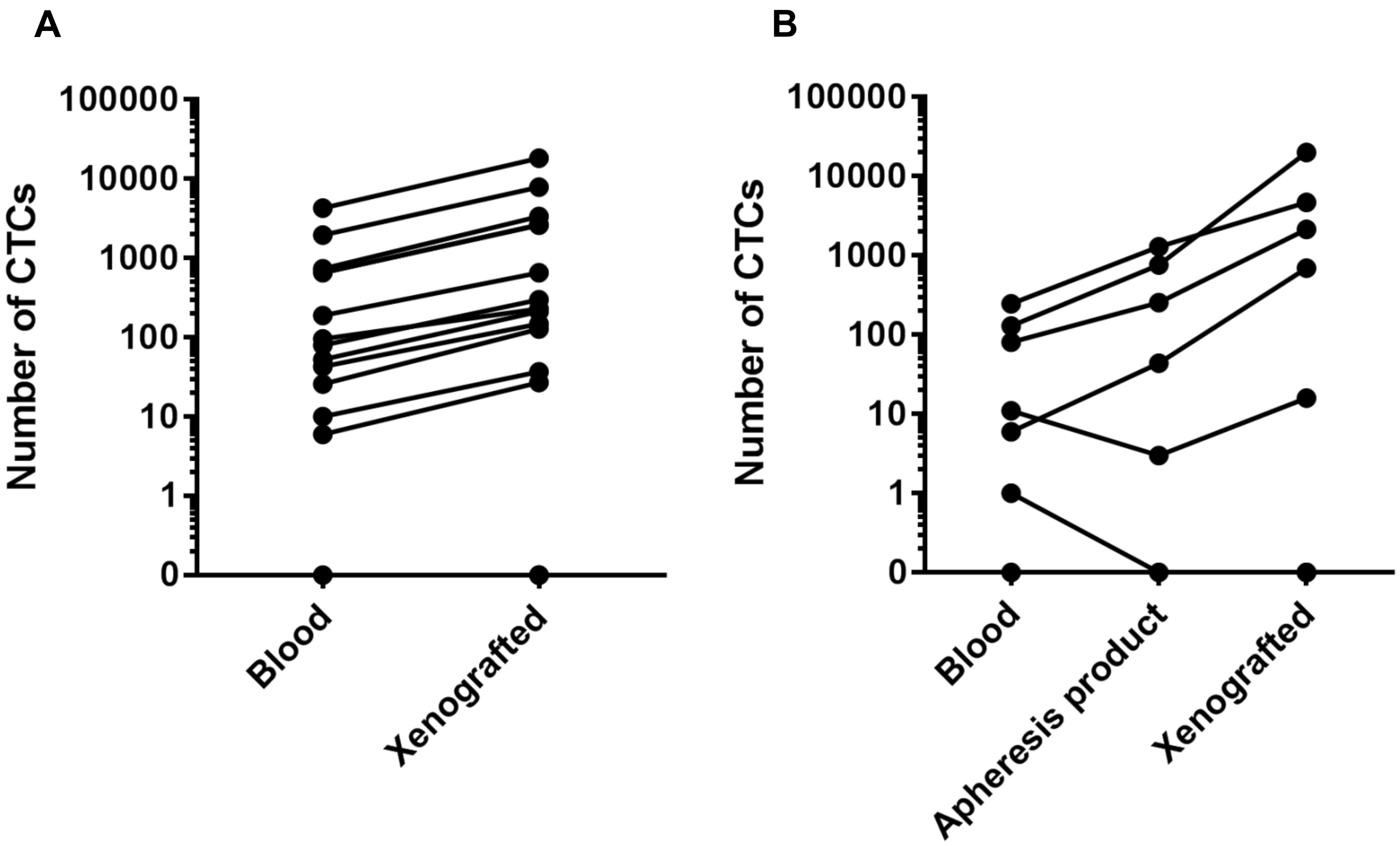

Supplementary Figure 1: CTC counts in blood samples and DLA products. (A), CTC counts in 7.5 ml blood and extrapolation of the numbers of xenografted CTCs based on CellSearch counts from patients with peripheral blood sampling only. Lines connect measurements from the same patient. **(B),** CTC counts in 7.5 ml blood, $200x10^6$ cells of DLA products and extrapolation of the numbers of xenografted CTCs based on CellSearch counts in DLA products from patients who underwent DLA. Lines connect measurements from the same patient.

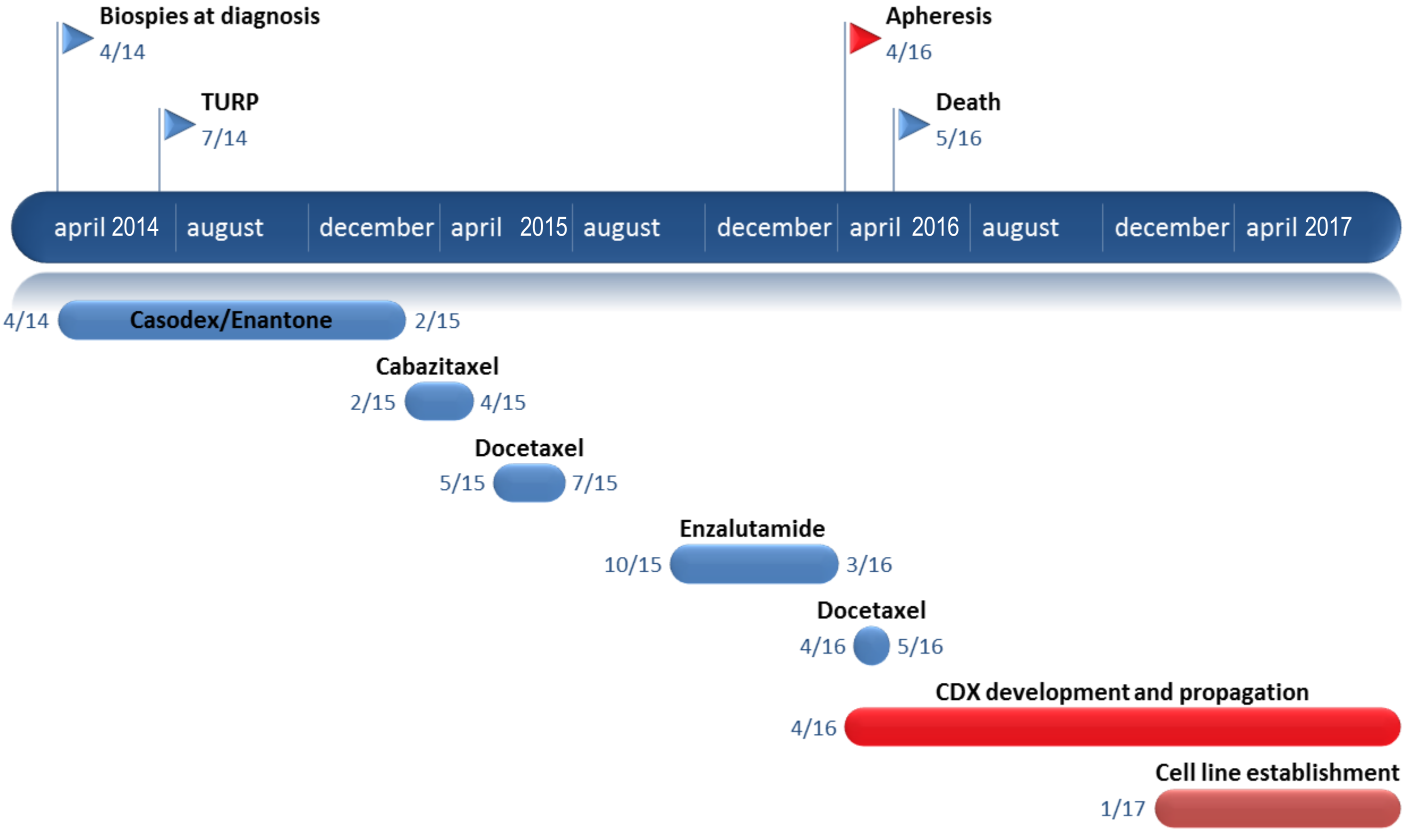

Supplementary Figure 2: Timelines of treatments, tumor and blood sample collection in Patient 3. Patient 3 underwent prostate biopsies and transurethral resection of prostate (TURP) on April 14th and July 17th 2014 respectively. On April 7th 2016 the DLA was performed following disease progression on enzalutamide. CTCs isolated from the DLA product were implanted into an NSG mouse leading to a palpable tumor within 165 days after implantation. The CDX was then propagated in successive generation of NSG mice. The cell line was established by sub culturing dissociated CDX tumor cells.

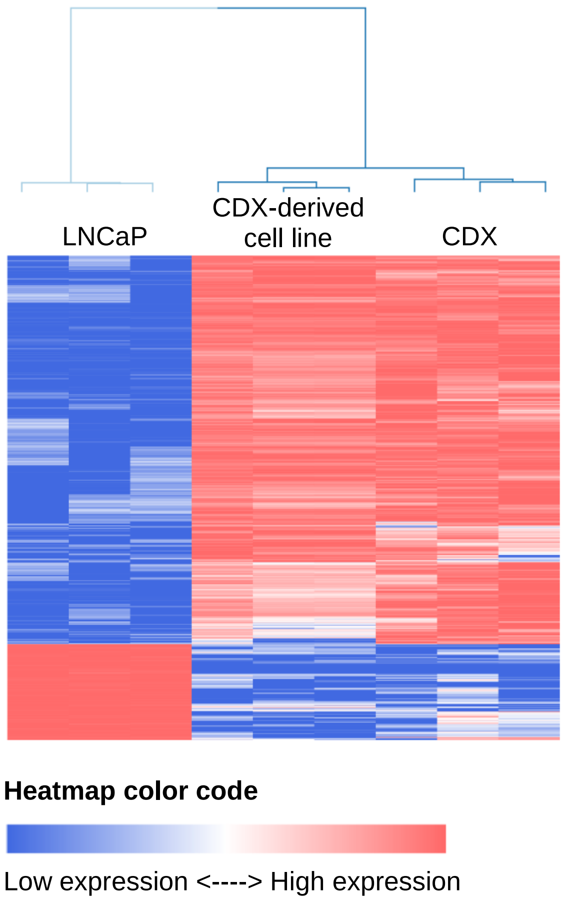

Supplementary Figure 3: Unsupervised hierarchical clustering of transcriptional profiles of triplicate samples of the LNCaP cell line, the CDX and the CDX-derived cell line. The rows show the normalized expression of the 1000 most variant genes (based on standard deviation) used to classify the samples according to their gene expression patterns.

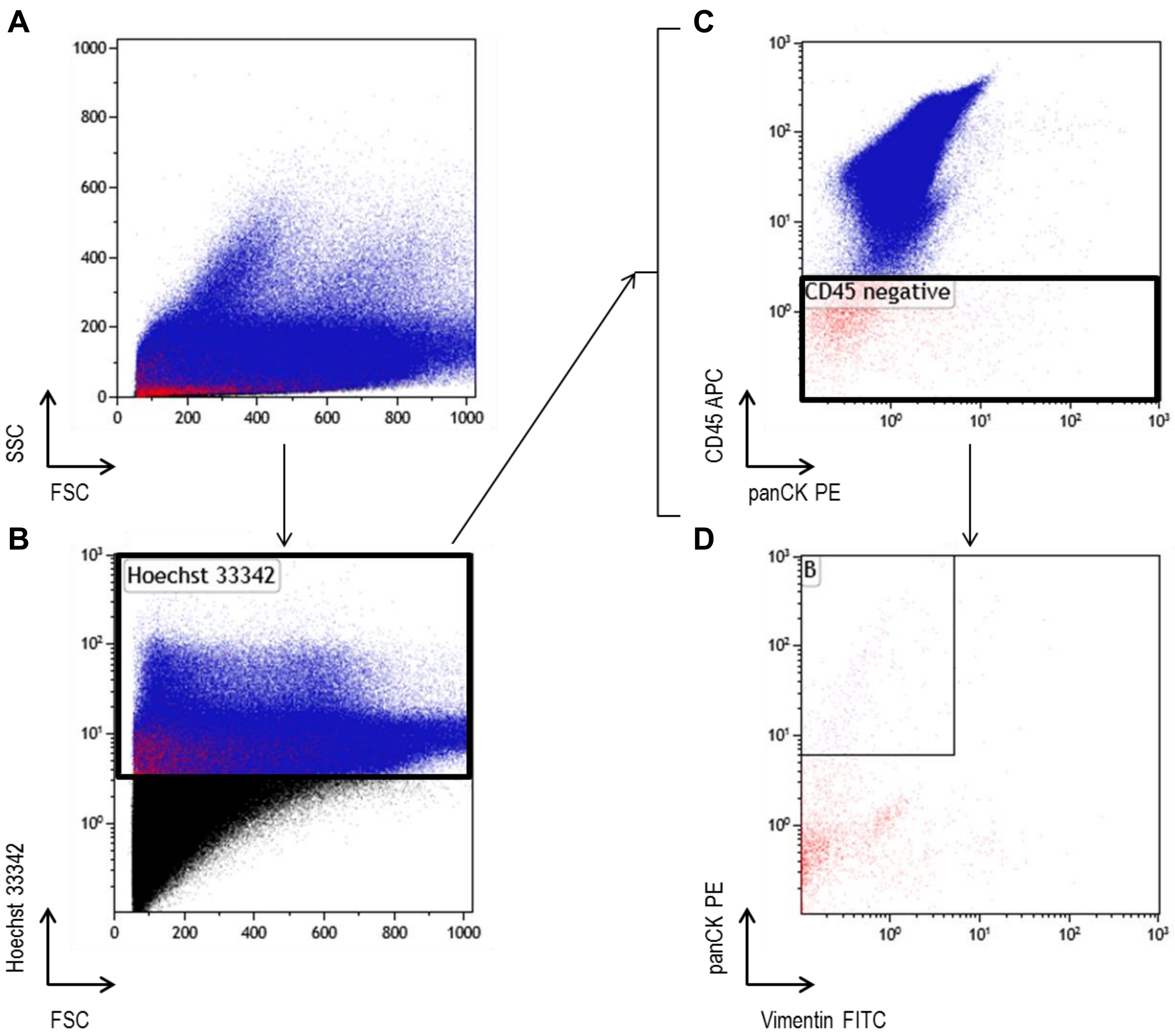

Supplementary Figure 4: Isolation of CTCs from Patient 3 DLA product by fluorescence activated cell sorting. (**A**), Cell selection according to size and granularity. (**B**), Selection of Hoecht 33342-positive elements according to size. (**C**), Selection CD45 negative and pan-cytokeratin positive cells. (**D**), Selection of Hoecht 33342-positive, CD45-negative, pan-cytokeratin positive CTCs.

A

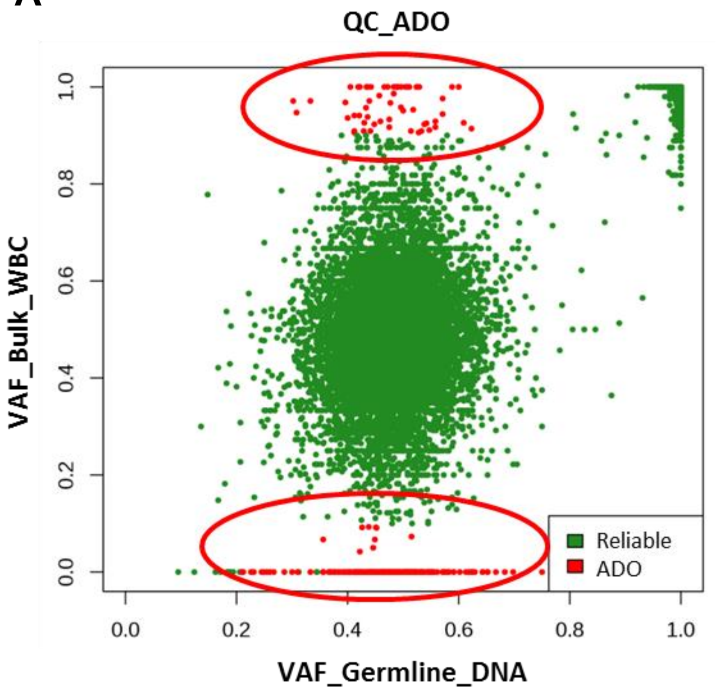

Proportion of germline variants with ADO
0.012648147

B

Sample	Number of false positive variants	Number of target bases covered ≥8X	False positive rate
CTC-1	236	33651882	7.01E-06
CTC-2	612	29475505	2.0763E-05
CTC-3	351	34740902	1.01034E-05
CTC-4	290	31112974	9.32087E-06
CTC-5	352	29528940	1.19205E-05
CTC-6	393	28570237	1.37556E-05

Supplementary Figure 5: Statistics of allele drop out and false positive rate of Patient 3 CTC samples. (A), Representation of allelic drop out (ADO). Reliable variants, in green, were defined by an equal VAF in both germline DNA and WBC bulk samples. Variants in ADO, in red, were defined by a VAF ranging from 0.2 to 0.8 in germline DNA and <0.1 or >0.9 in WBC bulk. **(B),** False positive rate in the six CTC samples. To estimate the false positive rates we divided the number of reliable somatic variants not present in bulk tumor samples (PTs and the CDX) by the number of target bases covered ≥8X in the same sample.

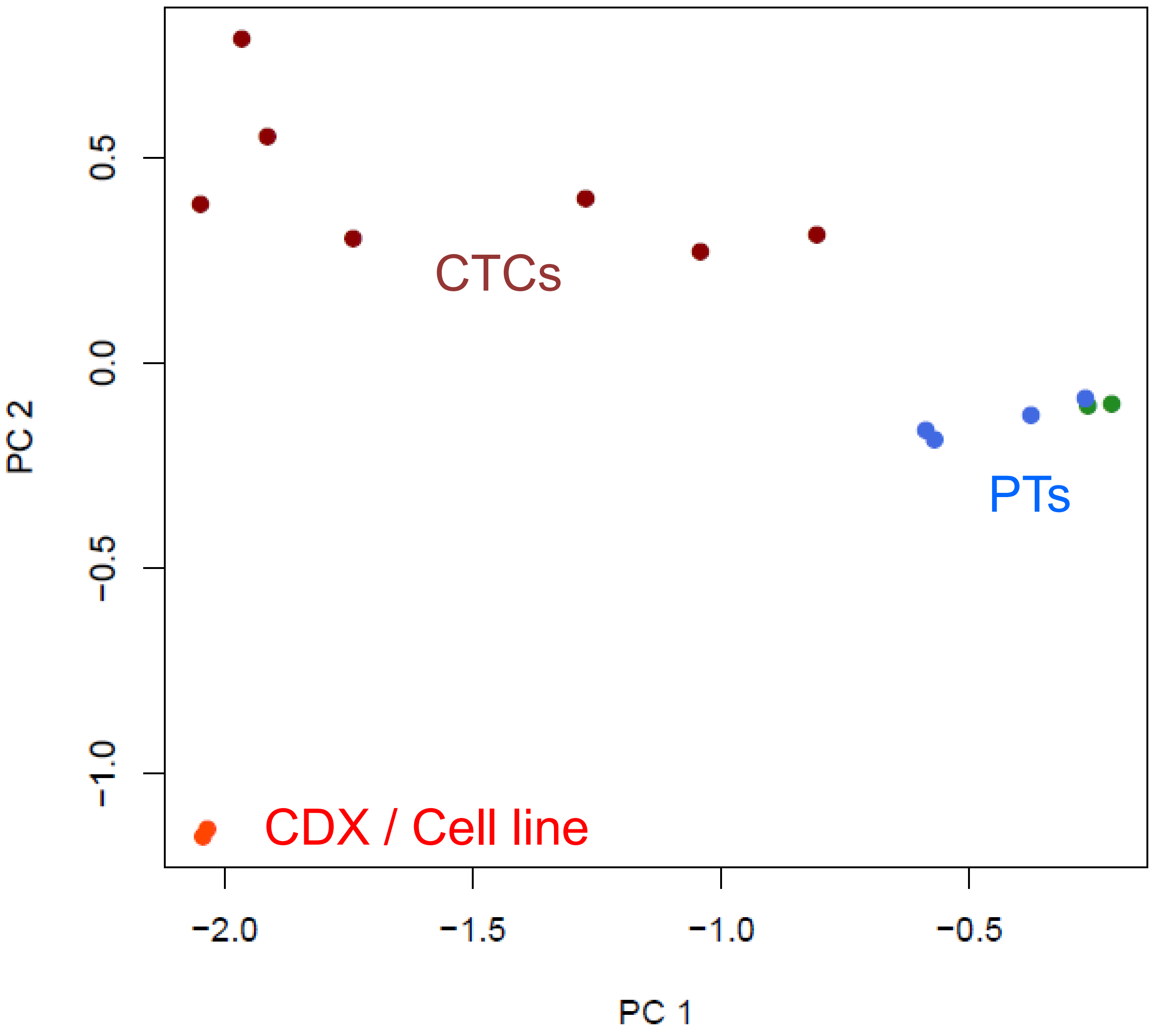

Supplementary Figure 6: Principal components analysis. To identify samples with similar mutational profiles, all variants present in at least two samples were selected and classified based on their VAF across these mutations using principal component analysis and hierarchical clustering (Ward method, cosine distance). This method allows to regroup samples sharing the same mutational profile. Three clusters were observed, one gathering all PTs, the second all CTC samples and the third the CDX and the cell line.

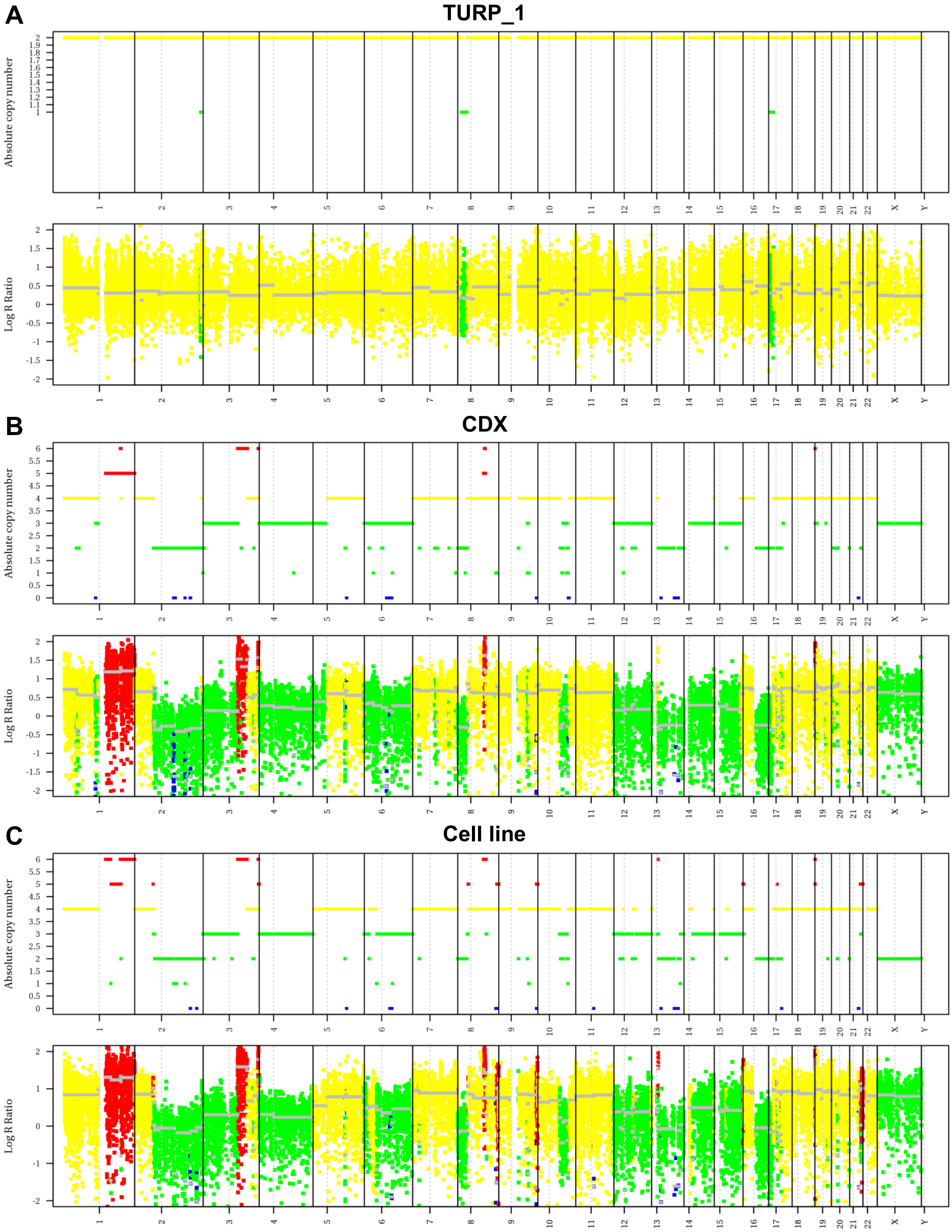
A
TURP_1
B
CDX
C
Cell line
Absolute copy number
Log R Ratio
1
2
3
4
5
6
7
8
9
10
11
12
13
14
15
16
17
18
19
20
21
22
X
Y

Supplementary Figure 7: Absolute copy number and log R ratio (LRR) profiles of TURP_1 (A), CDX (B) and the cell line (C). In each sample, yellow represented the normal/major copy number/LRR, red represented gains, green represented loss and blue represented homozygous deletion.

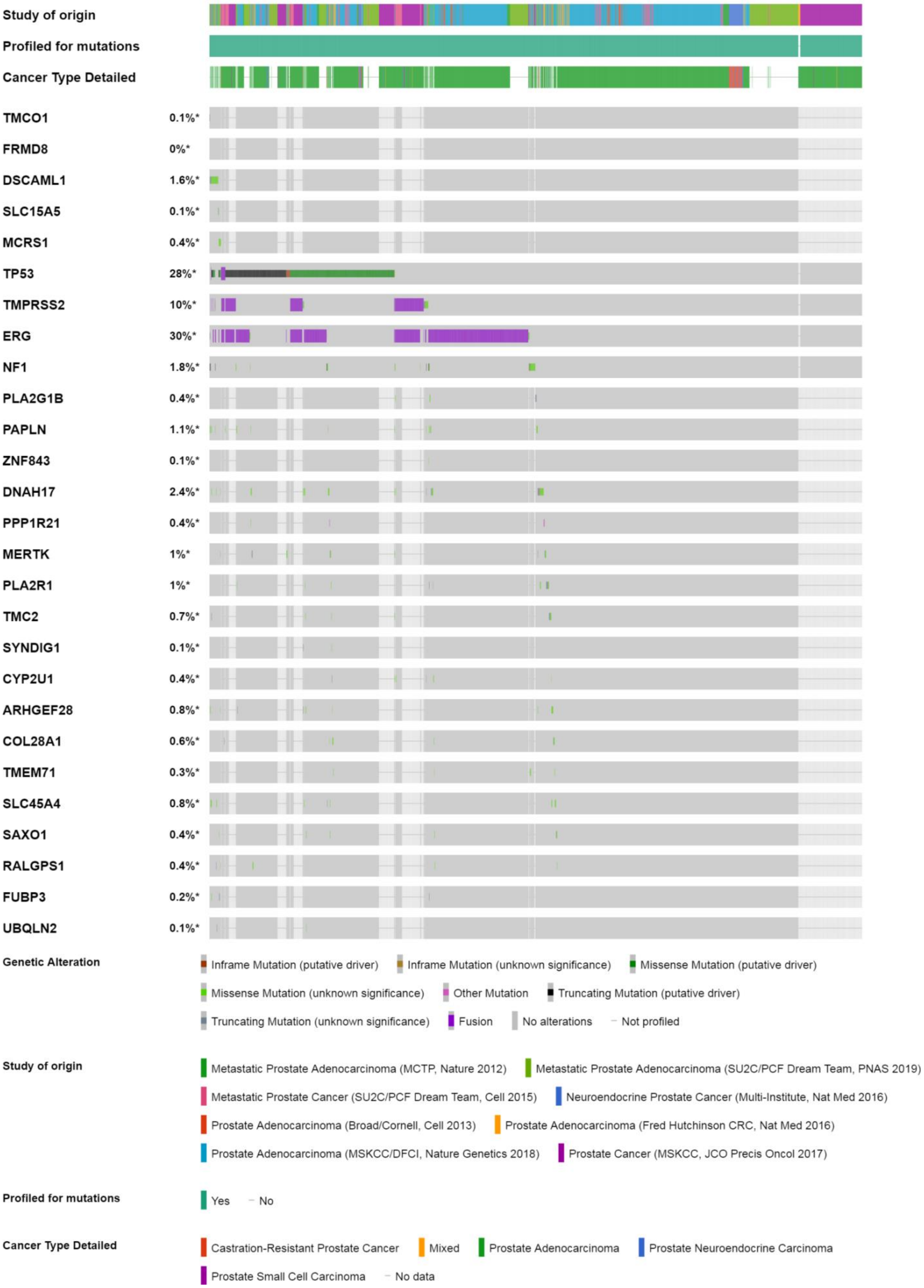
Study of origin
Profiled for mutations
Cancer Type Detailed
TMCO1 0.1%*
FRMD8 0%*
DSCAML1 1.6%*
SLC15A5 0.1%*
MCRS1 0.4%*
TP53 28%*
TMPRSS2 10%*
ERG 30%*
NF1 1.8%*
PLA2G1B 0.4%*
PAPLN 1.1%*
ZNF843 0.1%*
DNAH17 2.4%*
PPP1R21 0.4%*
MERTK 1%*
PLA2R1 1%*
TMC2 0.7%*
SYNDIG1 0.1%*
CYP2U1 0.4%*
ARHGEF28 0.8%*
COL28A1 0.6%*
TMEM71 0.3%*
SLC45A4 0.8%*
SAXO1 0.4%*
RALGPS1 0.4%*
FUBP3 0.2%*
UBQLN2 0.1%*
Genetic Alteration
Inframe Mutation (putative driver)
Inframe Mutation (unknown significance)
Missense Mutation (putative driver)
Missense Mutation (unknown significance)
Other Mutation
Truncating Mutation (putative driver)
Truncating Mutation (unknown significance)
Fusion
No alterations
Not profiled
Study of origin
Metastatic Prostate Adenocarcinoma (MCTP, Nature 2012)
Metastatic Prostate Adenocarcinoma (SU2C/PCF Dream Team, PNAS 2019)
Metastatic Prostate Cancer (SU2C/PCF Dream Team, Cell 2015)
Neuroendocrine Prostate Cancer (Multi-Institute, Nat Med 2016)
Prostate Adenocarcinoma (Broad/Cornell, Cell 2013)
Prostate Adenocarcinoma (Fred Hutchinson CRC, Nat Med 2016)
Prostate Adenocarcinoma (MSKCC/DFCI, Nature Genetics 2018)
Prostate Cancer (MSKCC, JCO Precis Oncol 2017)
Profiled for mutations
Yes
No
Cancer Type Detailed
Castration-Resistant Prostate Cancer
Mixed
Prostate Adenocarcinoma
Prostate Neuroendocrine Carcinoma
Prostate Small Cell Carcinoma
No data

Supplementary Figure 8: Percentage of CDX trunk genetic alterations (SNVs/INDELs) in **8 cBioPortal studies.** The percentages of samples harboring trunk genetic alterations in each gene are presented. The 8 interrogated studies [1-8] gathered 2,604 prostate tumor samples.

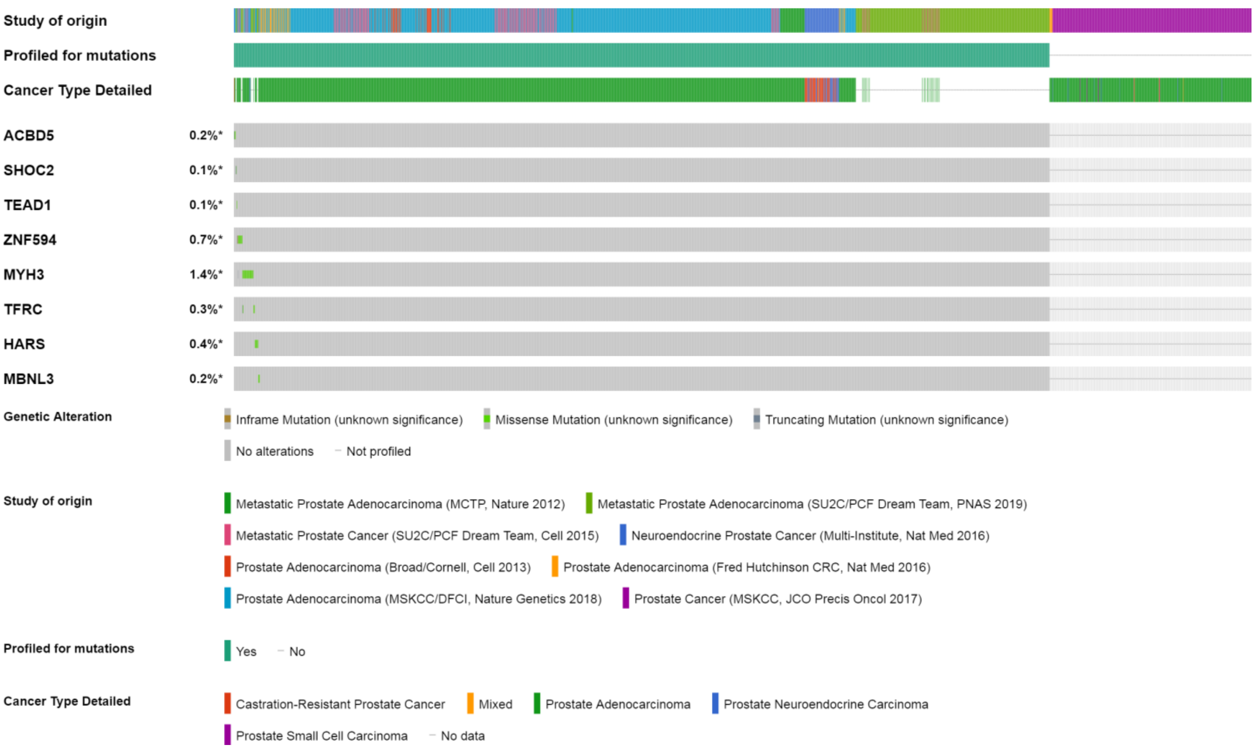

Supplementary Figure 9: Percentage of SNVs/INDELs acquired by the CDX in 8 cBioPortal studies. The percentages of samples harboring SNVs/INDELs in each gene are presented. The 8 interrogated studies [1-8] gathered 2,604 prostate tumor samples.

A

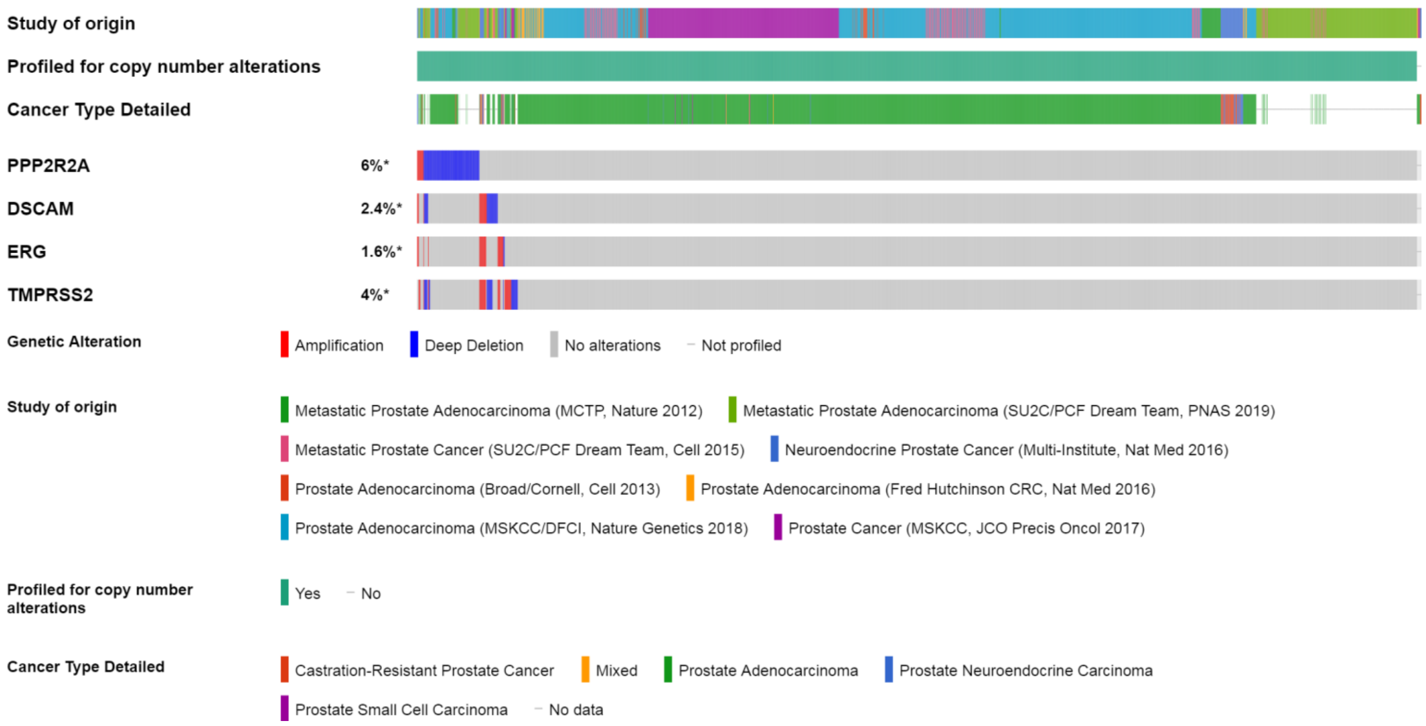
Study of origin
Profiled for copy number alterations
Cancer Type Detailed
PPP2R2A 6%*
DSCAM 2.4%*
ERG 1.6%*
TMPRSS2 4%*
Genetic Alteration
Amplification
Deep Deletion
No alterations
Not profiled
Study of origin
Metastatic Prostate Adenocarcinoma (MCTP, Nature 2012)
Metastatic Prostate Adenocarcinoma (SU2C/PCF Dream Team, PNAS 2019)
Metastatic Prostate Cancer (SU2C/PCF Dream Team, Cell 2015)
Neuroendocrine Prostate Cancer (Multi-Institute, Nat Med 2016)
Prostate Adenocarcinoma (Broad/Cornell, Cell 2013)
Prostate Adenocarcinoma (Fred Hutchinson CRC, Nat Med 2016)
Prostate Adenocarcinoma (MSKCC/DFCI, Nature Genetics 2018)
Prostate Cancer (MSKCC, JCO Precis Oncol 2017)
Profiled for copy number alterations
Yes
No
Cancer Type Detailed
Castration-Resistant Prostate Cancer
Mixed
Prostate Adenocarcinoma
Prostate Neuroendocrine Carcinoma
Prostate Small Cell Carcinoma
No data

B

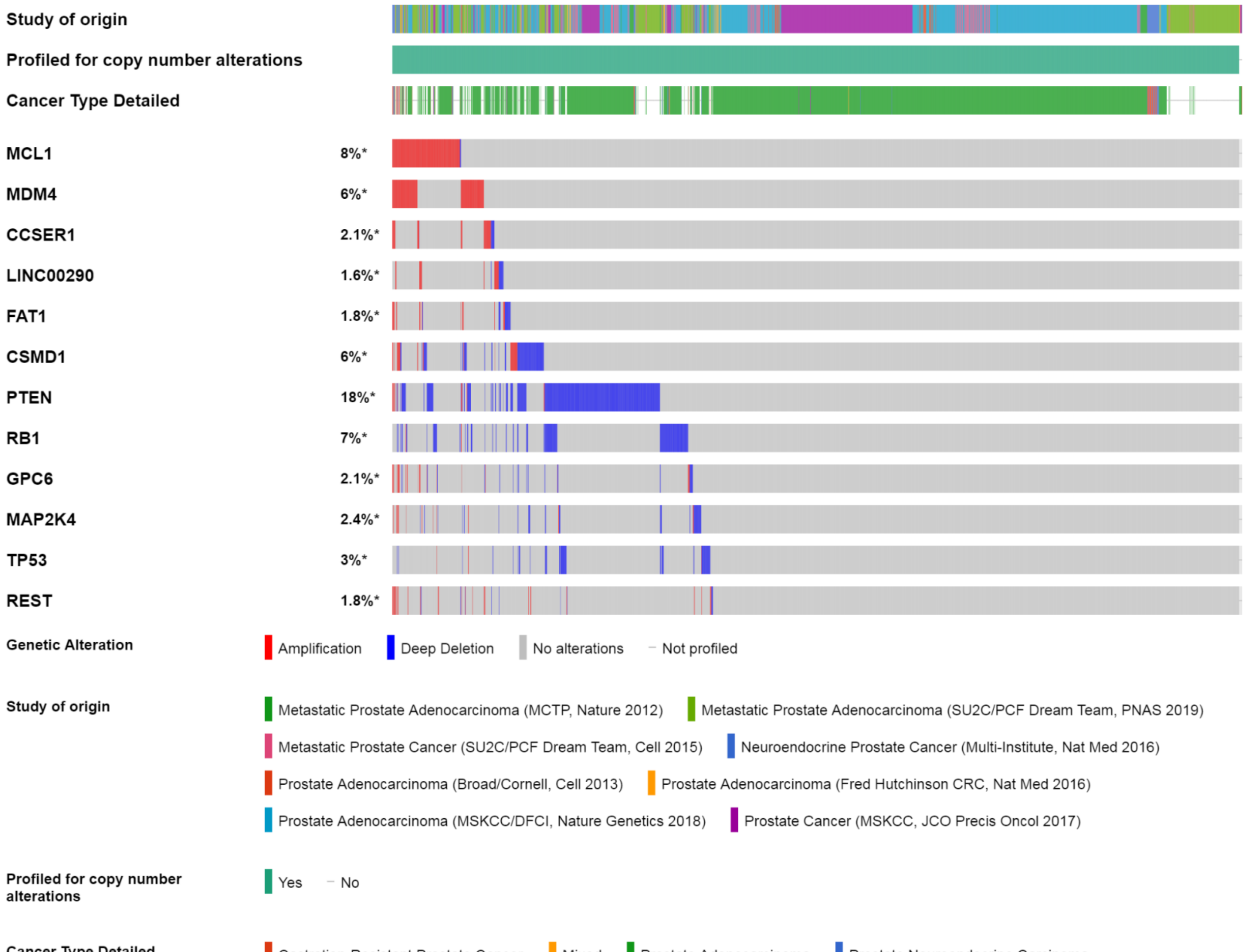
Study of origin
Profiled for copy number alterations
Cancer Type Detailed
MCL1 8%*
MDM4 6%*
CCSER1 2.1%*
LINC00290 1.6%*
FAT1 1.8%*
CSMD1 6%*
PTEN 18%*
RB1 7%*
GPC6 2.1%*
MAP2K4 2.4%*
TP53 3%*
REST 1.8%*
Genetic Alteration
Amplification
Deep Deletion
No alterations
Not profiled
Study of origin
Metastatic Prostate Adenocarcinoma (MCTP, Nature 2012)
Metastatic Prostate Adenocarcinoma (SU2C/PCF Dream Team, PNAS 2019)
Metastatic Prostate Cancer (SU2C/PCF Dream Team, Cell 2015)
Neuroendocrine Prostate Cancer (Multi-Institute, Nat Med 2016)
Prostate Adenocarcinoma (Broad/Cornell, Cell 2013)
Prostate Adenocarcinoma (Fred Hutchinson CRC, Nat Med 2016)
Prostate Adenocarcinoma (MSKCC/DFCI, Nature Genetics 2018)
Prostate Cancer (MSKCC, JCO Precis Oncol 2017)
Profiled for copy number alterations
Yes
No

Supplementary Figure 10: Percentage of CDX truncal or acquired CNAs in 8 cBioPortal studies. Genes bearing trunk **(A)** or acquired **(B)** CNAs were analyzed according to the 8 studies. The percentages of samples harboring truncal or acquired CNAs or each gene are reported. The 8 interrogated studies [1-8] gathered 2,604 prostate tumor samples.

A

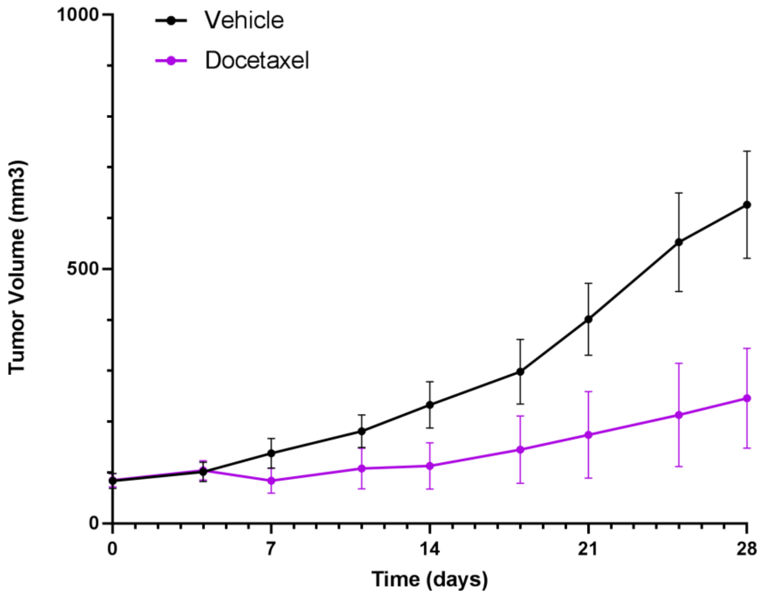

Vehicle
Docetaxel
Tumor Volume (mm3)
1000
500
0
0
7
14
21
28
Time (days)

B

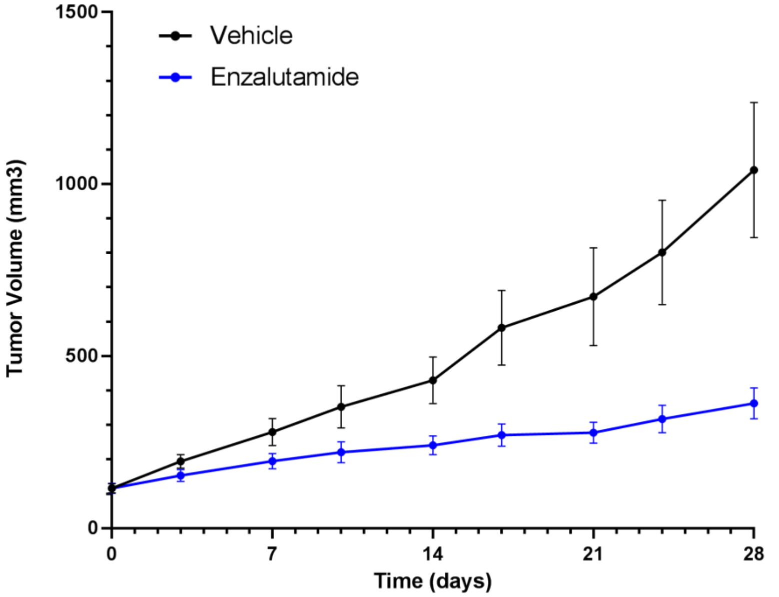

Vehicle
Enzalutamide
Tumor Volume (mm3)
1500
1000
500
0
0
7
14
21
28
Time (days)

C

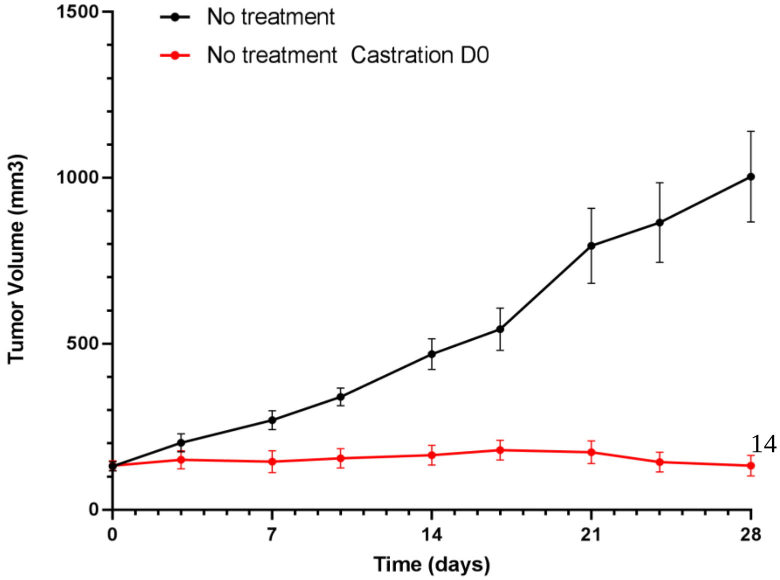

No treatment
No treatment Castration D0
Tumor Volume (mm3)
1500
1000
500
0
0
7
14
21
28
Time (days)

Supplementary Figure 11: *In vivo* drug assays performed using the PAC120 xenograft model. PAC120 is sensitive to docetaxel (**A**), enzalutamide (**B**) and castration (**C**). Non-castrated mice bearing tumors were treated with docetaxel, enzalutamide or the vehicle and the tumor volumes were monitored. A control group of mice receiving the vehicle was surgically castrated. In each group n=10 animals. Tumor volumes of vehicle and treated-groups over time after randomization are shown. Data represent mean tumor volumes ± s.e.m.

Supplementary Tables

Supplementary Table 1 : List of antibodies used for IHC and staining conditions

Antibodies	Manufacturer	Reference	Clone	Species	Dilution	Antigen retrieval
AR	Cell signaling	#5153	D6F11	Rabbit	1/50	40 minutes
CD44	Thermo Scientific	MS-668-P	156-3C11	Mouse	1/300	20 minutes
Chromogranin A	DAKO	#M0869	DAK-A3	Mouse	1/50	40 minutes
CK7	DAKO	#M7018	OV-TL-12/30	Mouse	1/50	40 minutes
CK8.18	Novocastra	#NCL-L-CK5/6/8/18	5D3, LP34	Mouse	1/100	40 minutes
Epcam	Cell signaling	#2929S	VU1D9	Mouse	1/500	40 minutes
Ki67	DAKO	#M7240	MIB-1	Mouse	1/50	40 minutes
NSE	DAKO	#M0873	BBS/NC/VI-H14	Mouse	1/100	40 minutes
PSA	DAKO	#M075029-2	ER-PR8	Mouse	1/50	40 minutes
Synaptophysin	DAKO	#M7315	DAK-SYNAP	Mouse	1/16	60 minutes
Vimentin	Santa Cruz	#SC-6260	V9	Mouse	1/500	no

Supplementary Table 2: List of antibodies used for FACS analyses

Antibodies	Manufacturer	Reference	Clone	Species	Fluorochrome	Dilution	Isotypes
EpCAM	BD Pharmingen	347200	EBA-1	Mouse	APC	1/20	Ms IgG1
CD133-2	Miltenyi	130-098-046	293C3	Mouse	PE	1/10	Ms IgG2b
CD166	R&D system	FAB6561P	105902	Mouse	PE	1/100	Ms IgG1
Pan-cytokeratins	ebioscience	53-9003-82	AE1/AE3	Mouse	AF488	1/100	Ms IgG1
E-cadherin	BD Pharmingen	560061	36/E-Cadherin	Mouse	AF488	1/200	Ms IgG2a

Supplementary Table 3: Number of variants identified in PT specimens, the CDX and the CDX-derived cell line.

Sample	Mean Depth	Median Depth	Coverage above 25X	SNVs	INDELs	SNVs + INDELs
TURP_1	130	98	89	24	22	46
TURP_2	130	104	90	40	23	63
Biopsy_1	81	46	80	40	17	57
Biopsy_2	144	72	86	26	17	43
Biopsy_3	149	72	85	30	13	43
Biopsy_4	98	61	86	12	3	15
Biopsy_5	133	69	85	23	20	43
Biopsy_6	130	67	85	36	30	66
CDX	109	80	81	72	8	80
Cell line	123	91	83	77	8	85

Supplementary Table 4: Number of high-confidence variants identified in CTC samples.

Sample	Mean Depth	Median Depth	Coverage above 25X	SNVs shared by at least 2 CTCs	SNVs shared by at least 1 CTC and 1 PT	SNVs shared by at least 1 CTC and the CDX/Cell line	High-confidence SNVs*	INDELs shared by at least 2 CTCs	INDELs shared by at least 1 CTC and 1 PT	INDELs shared by at least 1 CTC and the CDX/Cell line	High-confidence INDELs**	High-confidence SNVs + INDELs
CTC-1	88	23	51	21	10	11	22	6	1	2	6	28
CTC-2	90	14	45	24	11	13	25	10	3	4	10	35
CTC-3	88	26	53	23	13	15	24	4	2	3	4	28
CTC-4	87	18	48	20	11	13	23	3	2	3	3	26
CTC-5	102	15	46	26	13	17	27	10	2	4	10	37
CTC-6	93	12	44	20	12	17	23	8	3	5	8	31

* High-confidence SNVs were obtained by adding SNVs shared by at least 2 CTCs, SNVs shared by at least 1 CTC and 1 PT, SNVs shared by 1 CTC and the CDX/Cell line, Duplicates were considered only once.

** High-confidence INDELs were obtained by adding INDELs shared by at least 2 CTCs, INDELs shared by at least 1 CTC and 1 PT, INDELs shared by 1 CTC and the CDX/Cell line, Duplicates were considered only once.

References

1 Beltran, H. *et al.* Divergent clonal evolution of castration-resistant neuroendocrine prostate cancer. *Nat Med* **22**, 298-305, doi:10.1038/nm.4045 (2016).

2 Grasso, C. S. *et al.* The mutational landscape of lethal castration-resistant prostate cancer. *Nature* **487**, 239-243, doi:10.1038/nature11125 (2012).

3 Baca, S. C. *et al.* Punctuated evolution of prostate cancer genomes. *Cell* **153**, 666-677, doi:10.1016/j.cell.2013.03.021 (2013).

4 Abida, W. *et al.* Prospective Genomic Profiling of Prostate Cancer Across Disease States Reveals Germline and Somatic Alterations That May Affect Clinical Decision Making. *JCO Precis Oncol* **2017**, doi:10.1200/PO.17.00029 (2017).

5 Armenia, J. *et al.* The long tail of oncogenic drivers in prostate cancer. *Nat Genet* **50**, 645-651, doi:10.1038/s41588-018-0078-z (2018).

6 Kumar, A. *et al.* Substantial interindividual and limited intraindividual genomic diversity among tumors from men with metastatic prostate cancer. *Nat Med* **22**, 369-378, doi:10.1038/nm.4053 (2016).

7 Robinson, D. *et al.* Integrative clinical genomics of advanced prostate cancer. *Cell* **161**, 1215-1228, doi:10.1016/j.cell.2015.05.001 (2015).

8 Abida, W. *et al.* Genomic correlates of clinical outcome in advanced prostate cancer. *Proc Natl Acad Sci U S A* **116**, 11428-11436, doi:10.1073/pnas.1902651116 (2019).
