# Supplementary material for: Genetic characterization of a unique neuroendocrine transdifferentiation prostate circulating tumor cell-derived eXplant model
Source: Nat Commun. 2020 Apr 20;11:1884. doi: 10.1038/s41467-020-15426-2 (PMC7171138; doi:10.1038/s41467-020-15426-2)
Supplement: Supplementary file 3 — Description of Additional Supplementary Files [file 41467_2020_15426_MOESM3_ESM.pdf]

Description of Additional Supplementary Files

File Name: Supplementary Data 1

Description: List of the 250 functional genes that are relevant for CRPC-NE progression and significantly deregulated in the CDX and the CDX-derived cell line compared to LNCaP cells. Log2 fold change, pvalue and qvalue are presented for each gene.

File Name: Supplementary Data 2

Description: Description of all reliable somatic variants in PTs, CTCs, the CDX and the CDX-derived cell line.
