# Supplementary material for: Genetic characterization of a unique neuroendocrine transdifferentiation prostate circulating tumor cell-derived eXplant model
Source: Nat Commun. 2020 Apr 20;11:1884. doi: 10.1038/s41467-020-15426-2 (PMC7171138; doi:10.1038/s41467-020-15426-2)
Supplement: Supplementary file 6 — Reporting Summary [file 41467_2020_15426_MOESM6_ESM.pdf]

nature research

Corresponding author(s): Françoise Farace
NCOMMS-19-01047B

Last updated by author(s): Jan 15, 2020

Reporting Summary

Nature Research wishes to improve the reproducibility of the work that we publish. This form provides structure for consistency and transparency in reporting. For further information on Nature Research policies, see Authors & Referees and the Editorial Policy Checklist .

Statistics

For all statistical analyses, confirm that the following items are present in the figure legend, table legend, main text, or Methods section.

n/a	Confirmed	
☐	☒	The exact sample size (*n*) for each experimental group/condition, given as a discrete number and unit of measurement
☐	☒	A statement on whether measurements were taken from distinct samples or whether the same sample was measured repeatedly
☐	☒	The statistical test(s) used AND whether they are one- or two-sided<br>*Only common tests should be described solely by name; describe more complex techniques in the Methods section.*
☒	☐	A description of all covariates tested
☒	☐	A description of any assumptions or corrections, such as tests of normality and adjustment for multiple comparisons
☐	☒	A full description of the statistical parameters including central tendency (e.g. means) or other basic estimates (e.g. regression coefficient) AND variation (e.g. standard deviation) or associated estimates of uncertainty (e.g. confidence intervals)
☐	☒	For null hypothesis testing, the test statistic (e.g. F, t, r) with confidence intervals, effect sizes, degrees of freedom and P value noted<br>*Give P values as exact values whenever suitable.*
☒	☐	For Bayesian analysis, information on the choice of priors and Markov chain Monte Carlo settings
☒	☐	For hierarchical and complex designs, identification of the appropriate level for tests and full reporting of outcomes
☒	☐	Estimates of effect sizes (e.g. Cohen's d, Pearson's r), indicating how they were calculated

Our web collection on statistics for biologists contains articles on many of the points above.

Software and code

Policy information about availability of computer code

Data collection	Collection of FACS data was performed using the BD FACS Diva software v6.1.3. Collection of CellSearch data was performed using the Celltracks analyzer II.
Data analysis	- FACS data were analyzed using the Kaluza software (1.5a, Beckman Coulter). CTC analysis by Cellsearch was performed using Celltracks analyzer II.<br>- Bioinformatic analysis of WES was performed using Illumina Real-Time Analysis software sequence pipeline (2.7.7), Cutadapt (1.14), bamcmp (https://github.com/CRUKMI-ComputationalBiology/bamcmp), Burrows-Wheeler Aligner (BWA) tool (0.7.15-r1140), Sambamba (0.6.5), Broad Institute's GATK Haplotype Caller GVCF tool (3.7), MuTect2 tool (2.0), Bam-readcount (https://github.com/genome/bam-readcount), Ensembl's Variant Effect Predictor (VEP, release 87), Cancer Genome Interpreter (https://www.cancergenomeinterpreter.org/home), Bioconductor package DNAcopy (1.50.1), Bioconductor stats package (3.4.1), The R Bioconductor package phangorn v2.3.1.<br>- Statistical analysis of in vitro and in vivo drug assays was performed using GraphPrism5 software.<br>- Bioinformatic analysis of RNA sequencing was performed using Illumina Real Time Analysis (3.4.4), STAR1, Gencode v26 database, Bioconductor DESeq package (1.28.0), Bioconductor ConsensusClusterPlus package (1.40.0), Bioconductor edgeR package (3.18.1), Bioconductor limma package (3.32.10).

For manuscripts utilizing custom algorithms or software that are central to the research but not yet described in published literature, software must be made available to editors/reviewers. We strongly encourage code deposition in a community repository (e.g. GitHub). See the Nature Research guidelines for submitting code & software for further information.

Data

Policy information about availability of data

All manuscripts must include a data availability statement. This statement should provide the following information, where applicable:

- Accession codes, unique identifiers, or web links for publicly available datasets
- A list of figures that have associated raw data
- A description of any restrictions on data availability

All data supporting the findings of this study are available within the article and its supplementary information files and from the corresponding author (FF) upon request.

Field-specific reporting

Please select the one below that is the best fit for your research. If you are not sure, read the appropriate sections before making your selection.

- [x] Life sciences
- [ ] Behavioural & social sciences
- [ ] Ecological, evolutionary & environmental sciences

For a reference copy of the document with all sections, see nature.com/documents/nr-reporting-summary-flat.pdf

Life sciences study design

All studies must disclose on these points even when the disclosure is negative.

Sample size	- 22 patients who had given an informed consent for blood or DLA sampling were recruited. No statistical methods were used to predetermine sample size. This cohort of patients (n=22) was sufficient for the exploratory analysis performed in this study.<br>- For in vivo drug assays, sixty five athymic nude mice were implanted with passage-8 CDX. When tumor volumes ranged from 60 to 200 mm3, 7 mice were allocated to five groups. This number of mice was sufficient to obtain groups with homogenous mean and median tumor volumes.
Data exclusions	- Due to the lower quality of collected material evidenced by DNA degradation, data of biopsies 1 and 4 were excluded from variant identification but conserved for detecting variants found in other PT specimens.<br>- For the mutation analysis, several filters were applied in order to generate high-confidence variants from CTCs. The filters applied are indicated in the Methods. They were established on the basis of previous publications.
Replication	- All attempts to replicate in vivo and in vitro studies were successful.<br>- RNA sequencing was performed in triplicate. Triplicate values were consistent and no data was excluded.
Randomization	- For in vivo studies, treatments were randomly attributed to the five different mice groups.<br>- There is no other experiments requiring randomization since the study was not comparing different treatments.
Blinding	The investigators were blinded to group allocation during data collection and analysis.

Reporting for specific materials, systems and methods

We require information from authors about some types of materials, experimental systems and methods used in many studies. Here, indicate whether each material, system or method listed is relevant to your study. If you are not sure if a list item applies to your research, read the appropriate section before selecting a response.

Materials & experimental systems

n/a	Involved in the study	
[ ]	[x]	Antibodies
[ ]	[x]	Eukaryotic cell lines
[x]	[ ]	Palaeontology
[ ]	[x]	Animals and other organisms
[ ]	[x]	Human research participants
[ ]	[x]	Clinical data

Methods

n/a	Involved in the study	
[x]	[ ]	ChIP-seq
[ ]	[x]	Flow cytometry
[x]	[ ]	MRI-based neuroimaging

Antibodies

Antibodies used	All the antibodies used in the study are described in detail in the supplementary Tables 1 and 2.<br>For IHC experiments:<br>AR Cell signaling #5153 D6F11 Rabbit<br>CD44 Thermo Scientific MS-668-P 156-3C11 Mouse

	Chromogranin A DAKO #M0869 DAK-A3 Mouse<br>CK7 DAKO #M7018 OV-TL-12/30 Mouse<br>CK8.18 Novocastra #NCL-L-CK5/6/8/18 5D3, LP34 Mouse<br>Epcam Cell signaling #2929S VU1D9 Mouse<br>Ki67 DAKO #M7240 MIB-1 Mouse<br>NSE DAKO #M0873 BBS/NC/VI-H14 Mouse<br>PSA DAKO #M075029-2 ER-PR8 Mouse<br>Synaptophysin DAKO #M7315 DAK-SYNAP Mouse<br>Vimentin Santa Cruz #SC-6260 V9 Mouse<br>For FACS analysis of the CDX-derived cell line:<br>EpCAM BD Pharmingen 347200 EBA-1 Mouse<br>CD133-2 Miltenyi 130-098-046 293C3 Mouse<br>CD166 R&D system FAB6561P 105902 Mouse<br>ALDH StemCell Technologies ALDEFLUOR Kit 01700<br>Pan-cytokeratins ebioscience 53-9003-82 AE1/AE3 Mouse<br>E-cadherin BD Pharmingen 560061 36/E-Cadherin Mouse<br>For cell sorting experiments: Cytokeratins-PE (cytokeratins 8, 18, 19) and CD45-APC antibodies of CellSearch CTC kit (Menarini Silicon Biosystems), Vimentin Santa Cruz #SC-6260 V9 Mouse conjugated to AF488
Validation	All antibodies have been validated using controls. For each batch, the optimal dilution has been determined.<br>For FACS analyses, The LNCaP cell line (ATCC) and blood mononuoclear cells were used for positive and negative controls of epithelial markers and vimentin respectively.<br>Positive control for CD166 was blood mononuclear cells.<br>Positivity of CD133 was tested on endothelial progenitors.<br>Positive and negative controls of ALDH activity were performed according to ALDEFLUOR Kit recommendations<br>IHC was performed manually using the coverplate system. Two protocols were developed depending of the species used to raise primary antibodies (mouse or rabbit) to avoid background due to mouse cells present in the CDX. Mouse Klear kit (GBI labs) and EnVision rabbit kit (Agilent Technologies, Dako) were used for primary mouse and rabbit antibodies respectively. Positive controls for each antibodies were as following:<br>AR: Cerebral vesicle tumor<br>CD44: Tumor ovarian adenocarcinoma<br>ChromograninA and Synaptophysin: Neuroendocrine tumor<br>CK7 and CK8.18: Non-tumor breast<br>EpCAM, KI67, NSE and Vimentin: Non-tumor appendix<br>PSA: Non-tumor prostate

Eukaryotic cell lines

Policy information about cell lines

Cell line source(s)	LnCAP cell line was purchased from the American Type Culture Collection (ATCC).
Authentication	No further authentication.
Mycoplasma contamination	All cell lines were tested negative for mycoplasma contamination.
Commonly misidentified lines (See ICLAC register)	No misidentified cell line was used in the study.

Animals and other organisms

Policy information about studies involving animals; ARRIVE guidelines recommended for reporting animal research

Laboratory animals	NOD.Cg-Prkdcscid Il2rgtm1Wjl/SzJ mice (NSG) 6 week old male mice (Charles River Laboratories, Wilmington, MA, USA) were used for circulating tumor cell implantation.<br>6-9 week-old, male athymic nude mice (Hsd:Athymic Nude-Fox1nu) provided by ENVIGO (Gannat, France) were used for in vivo drug assays.
Wild animals	The study did not involve wild animals.
Field-collected samples	The study did not involve samples collected from the field.
Ethics oversight	The study was approved by the French Ministry of Higher Education, Research and Innovation (Ministère de l'Enseignement supérieur, de la Recherche et de l'Innovation, MESRI).

Note that full information on the approval of the study protocol must also be provided in the manuscript.

Human research participants

Policy information about studies involving human research participants

Population characteristics	All the patient characteristics relevant for the study are presented in the Table 1 of the manuscript. The median age of patients undergoing DLA was 65. Two patients had prostate carcinoma and five a prostate adenocarcinoma. The tumor molecular status was determined only for Patient 3. All patients had castration, and/or enzalutamide and/or abiraterone and/or chemotherapy before apheresis.<br><br>Inclusion criteria:<br>- Patients with solid metastatic tumor.<br>- Patients with > 18 years of age.<br>- Written informed consent.<br>Specific inclusion criteria for DLA study:<br>- Recognized suitable for sampling by the collecting doctor after a medical examination including a measurement of blood pressure, heart rate and an electrocardiogram.<br>- Accepting the removal of mononuclear cells by cytapheresis for the purpose of scientific research.<br><br>Exclusion criteria:<br>- Patients protected by law, in accordance with articles L1121-5 to L1121-8 of the Public Health Code.<br>- Mental pathology which could interfere with the smooth running of the study.<br>- Not consenting.<br>Specific exclusion criteria for DLA study:<br>- In order to avoid any institutional constraint, people with some dependence on Gustave Roussy will not be able to participate in this study.<br>- HIV +, HTLV1 +, HCV +, HBV + serology (AgHBS + and / or isolated anti-HBc + Ab).<br>- History of epilepsy, tetany crisis, high blood pressure and heart abnormalities, asthma, chronic renal failure, obstructive pulmonary disease, bleeding disorder.<br>- total white blood cells less than 4000 / mm3.<br>- Anemia <11 g / dl.<br>- Thrombocytopenia <150,000.
Recruitment	The study (IDRCB2008-A00585-50) was conducted at Gustave Roussy (Villejuif, France). Consenting patients to undergo DLA were recruited according to the study eligibility criteria. No selection bias has been identified.
Ethics oversight	This study was authorized by the French national regulation agency ANSM (Agence Nationale de Sécurité du Medicament et des produits de santé), and approved by the Ethics Committee and our institutional review board. Informed written consent was obtained from all patients.

Note that full information on the approval of the study protocol must also be provided in the manuscript.

Clinical data

Policy information about clinical studies

All manuscripts should comply with the ICMJEguidelines for publication of clinical research and a completedCONSORT checklist must be included with all submissions.

Clinical trial registration	IDRCB2008-A00585-50
Study protocol	The IDRCB2008-A00585-50 full trial protocol can be accessed at the Department of Clinical Research, Institut Gustave Roussy (Villejuif, France).
Data collection	Patients were treated at Institut Gustave Roussy (IGR). Patient follow up and data (e. g. clinical characteristics, treatments) collection were performed between March 2014 and June 2016 at IGR.
Outcomes	The study was focused on CDX establishment and characterization. Outcomes of patients for whom a CDX establisment was unsuccessful were not relevant for the study and data were not collected. The clinical history of Patient 3 for whom a CDX was established is presented in the Supplementary Figure 2.

Flow Cytometry

Plots

Confirm that:

☒ The axis labels state the marker and fluorochrome used (e.g. CD4-FITC).

☒ The axis scales are clearly visible. Include numbers along axes only for bottom left plot of group (a 'group' is an analysis of identical markers).

☒ All plots are contour plots with outliers or pseudocolor plots.

☒ A numerical value for number of cells or percentage (with statistics) is provided.

Methodology

Sample preparation	Following RosetteSep enrichment, cells were washed with 1X PBS and centrifuged at 560g for 5 min. The pellet was then resuspended with 100 µl of fixative solution A of Fix&Perm kit (Thermo Fisher Scientific), washed with 1X PBS and centrifuged at 370g for 5 min. The pellet was resuspended in 100 µl of permeabilization solution medium B of Fix&Perm kit and 50 µl of a staining solution of cytokeratins-PE (cytokeratins 8, 18, 19) and CD45-APC antibodies of a CellSearch CTC kit (Menarini Silicon Biosystems) and 5 µl anti-vimentin-FITC antibody (clone V9, Santa Cruz Biotechnology) was added. The cell suspension was then incubated for 20 min at RT, washed with 1X PBS and centrifuged at 370g 1300 rpm for 5 min. The pellet was resuspended in 300 µl of 1X PBS and kept at +4°C. Hoechst was added before cell sorting.
Instrument	Cell sorting was performed using a BD FACSARIA III cell sorter (BD Biosciences) equipped with four lasers (a 405 nm laser, a 488 nm laser, a 561 nm laser and a 640 nm laser). The system was run with 20 psi pressure, a 100 µm nozzle and the yield precision mode. Acquisition of immunofluorescence data was performed with LSR Fortessa cytometer (BD Biosciences) equipped with BD FACS Diva software.
Software	Data were analyzed using the Kaluza Software (Beckman Coulter).
Cell population abundance	Pools of five single cells were sorted.
Gating strategy	Hoechst 33342-positive elements were first gated. The second gate enabled selection of CD45-APC negative events. CD45-APC-/CK-PE+/Vim-FITC- circulating tumor cells were sorted and collected.

☒ Tick this box to confirm that a figure exemplifying the gating strategy is provided in the Supplementary Information.
